# Animal welfare of Lacaune lambs weaned from artificial feeding

**DOI:** 10.3389/fvets.2024.1474801

**Published:** 2024-10-15

**Authors:** María Moreno Manrique, Carlos Mínguez Balaguer, Carla Ibáñez Sanchis, Marta González Clari, Arantxa Villagrá García, Joel Bueso Ródenas

**Affiliations:** ^1^Doctoral School, Department of Animal Production and Public Health, Faculty of Veterinary Medicine and Experimental Sciences, Catholic University of Valencia San Vicente Mártir, Valencia, Spain; ^2^Department of Animal Production and Public Health, Faculty of Veterinary Medicine and Experimental Sciences, Catholic University of Valencia San Vicente Mártir, Valencia, Spain; ^3^Valencian Institute for Agricultural Research | IVIA Center for Animal Research and Technology (CITA-IVIA), Segorbe, Castellón, Spain

**Keywords:** Lacaune lambs, milk replacer, animal behavior, dairy sheep, animal stress

## Abstract

**Introduction:**

Nowadays, many dairy sheep farms opt for milk replacers after birth. Weaning lambs from milk replacers is expected to be a stressful situation.

**Methods:**

With the aim of researching this practice on the animal behavior, body weight yields, and sanitary status of Lacaune lambs, 60 healthy animals from the same lambing house were employed. Lambs were housed in a pen and had *ad libitum* access to forage, compound feed, and milk replacers. During a 4 days preexperimental period in which all the animals were fed with milk replacers, behavior, hematologic parameters, body weight and seric and fecal stress indicators were recorded. Later, an experimental period took place in which 20 lambs remained in the same conditions. Another 20 lambs were kept in a separate pen in the same barn under the same conditions, but the artificial milk feeding was interrupted (weaning). The third 20 lambs were weaned and also rehoused in fattening pens. After 4 days, the variables previously recorded in the three groups during the pre-experimental period were recorded again.

**Results:**

Results showed that change of location and change of feed can have different and synergic effects on the behavior of the lambs. Change of feed had not specifically different effect on body weight than change of location. In the other hand, weaning had no significative effects on hematologic parameters and seric and fecal stress indicators.

**Discussion:**

Weaning from artificial milk had significative effects on lambs weight and behavior. More research is needed to improve this ethical aspect in ovine production.

## 1 Introduction

Dairy sheep farming plays an important role in the agricultural sector, making significant contributions to the production of high-quality milk and dairy products (1, 2). Lacaune breed ewes, well-known for their specialized milk production capabilities (3), are extensively used in dairy farms worldwide (4, 5). Nowadays, many dairy sheep farms opt for artificial milk or milk replacers after birth, while immediately providing natural colostrum to the lambs either directly or indirectly (6). This choice helps reduce the burden on ewes, enabling them to conserve energy for milk production. After weaning, lambs from dairy sheep farms may follow various paths, including going to slaughterhouses, being finished on the birth farm, placed in a lamb feedlot, or staying in the original farm to serve as adult breeding ewes (7).

The practice of weaning lambs from milk replacers as soon as possible to cut down on costs is expected to be a stressful situation, similar to lactation with the mother and early weaning. Several studies have investigated the impact of stress on lamb health and welfare under different scenarios, such as lactation with the mother, early weaning, and feedlot finishing. For instance, Miranda-de la Lama et al. (8) found that stay time at the classification center and season influenced stress variables. Miranda-de la Lama et al. (9) observed that exposure to novel environments and social mixing induced stress responses in lambs, evident by increased aggression, stereotypes, and plasma cortisol levels. In another study, Dalmau et al. (10) reported that longer transport times tended to increase cortisol and lactate levels. Additionally, Galapero et al. (11) indicated that the neutrophil/lymphocyte ratio, phagocytosis index, and cortisol levels serve as valuable indicators of stress conditions and the potential for diseases. Fernández et al. (12) also used Hematocrit, Cellular Hemoglobin Concentration and Cortisol levels to identify elevated acute stress in lambs during the feedlot period, transport, and handling. On the other hand, other studies have shown that the count of generic *E. coli* in feces can be an indicator of stress in animals (13–15).

Different strategies to feed lambs, including a combination of both natural and artificial milk (16), or early weaning strategies (17–19) aimed at increasing milk production in the mothers or reducing the time spent on milk replacers to approximately 30 days, are common. In the study by Belanche et al. (18), milk replacers facilitated the successful rearing of lambs, achieving similar productive parameters to lactation with the mother. However, both feeding with artificial milk and early weaning, like in lactation with the mother cases, present challenges for lamb welfare, sanitary status, and performance. Age of the weaned lambs has an effect on animal welfare. Two days lambs experienced more stress than 3 months lambs when both groups were separated from their mothers as their behavior patterns showed in the study of Napolitano et al. (17). Despite this, there are currently no studies in the literature monitoring these aspects specifically in weaned Lacaune lambs fed with milk replacers. Thus, the aim of this study was to investigate the effects of early weaning on the animal behavior, lamb weight yields, blood and serum parameters and count of *E. coli* colonies from feces of Lacaune lambs fed with milk replacers.

## 2 Materials and methods

The experiment carried out and included in this work has been approved by the Animal Experimentation Ethics Committee of the Catholic University of Valencia “San Vicente Mártir” (UCV), with reference code CEEAUCV2012.

### 2.1 Facilities and animal handling

The study was carried out during March 2022 at the La Muntanyeta Sociedad Cooperativa Valenciana dairy sheep farm, belonging to the Granja Rinya group located in Catadau/Llombai Valencia (Spain). It is a farm with a census of approximately 5,000 Lacaune sheep whose objective is milk production and the production of fattened lambs. The farm has mechanical milking facilities, separate rooms where lambs have access to *ad libitum* artificial milk, lambs fattening pens and a warehouse for raw materials for animal feed. The adult sheep are located in free stall barns stabling in 6 different parks depending on their productive state. Natural mating after heat synchronization using progesterone sponges (Chronogest, MSD Animal Health Spain, Carbajosa de la Sagrada, Spain) is practiced. After the births, the lambs are immediately separated from the mothers, they are identified, their umbilical scars are disinfected and colostrum is administered for 2 days using natural colostrum from the farm itself, frozen and tempered. After this period, the lambs are fed by milk replacers in 15 square meters surface pens with cereal straw beds in groups of approximately 60 animals in. The feeding of the lambs during the lactation period is based on milk replacer ELVOR 63 (crude protein: 24%; Fat: 24%; Cellulose: 5%; Ash 7%; Ca 0.9%; Na: 0.45%; P: 0.75%) administered *ad libitum* by JR milk replacer machine (JR, El Torno, Ciudad Real, Spain), cereal straw and compound feed (Lactoiniciacor Nanta, Silla, Valencia; Crude protein: 18%; Crude fiber: 4%; fat: 3%; ashes 6.9%, starch + sugars: 43%). Water is also offered *ad libitum*. After weaning the animals are transported from the milk replacer feeding rooms to 15 square meters surface fattening pens with cereal straw beds. The females form the future replacement and the males, except those that due to their genealogy are destined as stallion males, are destined for slaughter. The feeding of the animals in this phase are based on compound feed (Nantacor intensive bait Nanta, Silla, Valencia; Protein: 17.5%; Fiber: 4.3%; Fat: 3%; Ashes 42%, starch + sugars: 42%), cereal straw, and water *ad libitum*.

### 2.2 Experimental design and variables studied

For the study, 60 lambs from the same lambing house were used in a Randomized block design study. These lambs followed the usual handling of the farm above described. Specifically, 34 males and 26 females, 34 ± 3 days old and with an average weight of 16.4 ± 2.1 kg. For the selection of the animals included in the experiment, healthy animals with similar body condition were chosen, under the evaluation of a veterinary team, ruling out animals with reduced body condition, skin, ophthalmic, locomotor, respiratory or digestive problems. Animals were randomly divided in three similar groups of 20 animals according their sex and weight.

The pre-experimental period lasted 4 days, during which the 3 batches of animals were kept under conditions similar to those described above for the animals in lactation. The experimental period began on day 5 (day 0 of the experiment). During this period, the 3 batches of animals continued under different conditions (treatments) from each other. The allocation between batches and treatments to be studied was carried out randomly.

On day 0 of the experiment (lambs were 39 ± 3 days old) the first group remained in the same installations continuing with artificial milk feeding as prolonged lactation (Suckling group: SG), with animals of 16.5 kg of average weight, 9 females and 11 males. The second group was weaned (*ad libitum* administration of artificial milk was interrupted and animals were fed only with forage and compound feed) and stayed in the same installations (Weaned group: WG), with animals of 16.3 kg of average weight, 9 females and 11 males. And the third group was weaned and was rehoused in the fattening pens that are 200 m far from the lactation rooms, following the typical handling of the farm (Rehoused group: RG). The transfer of the lambs was gently done with a truck by the workers of the farm and supervised by the researchers, ensuring high standards of animal welfare. This group was made up of 8 females and 12 males, with an average weight of 16.5 kg.

**Figure 1:**
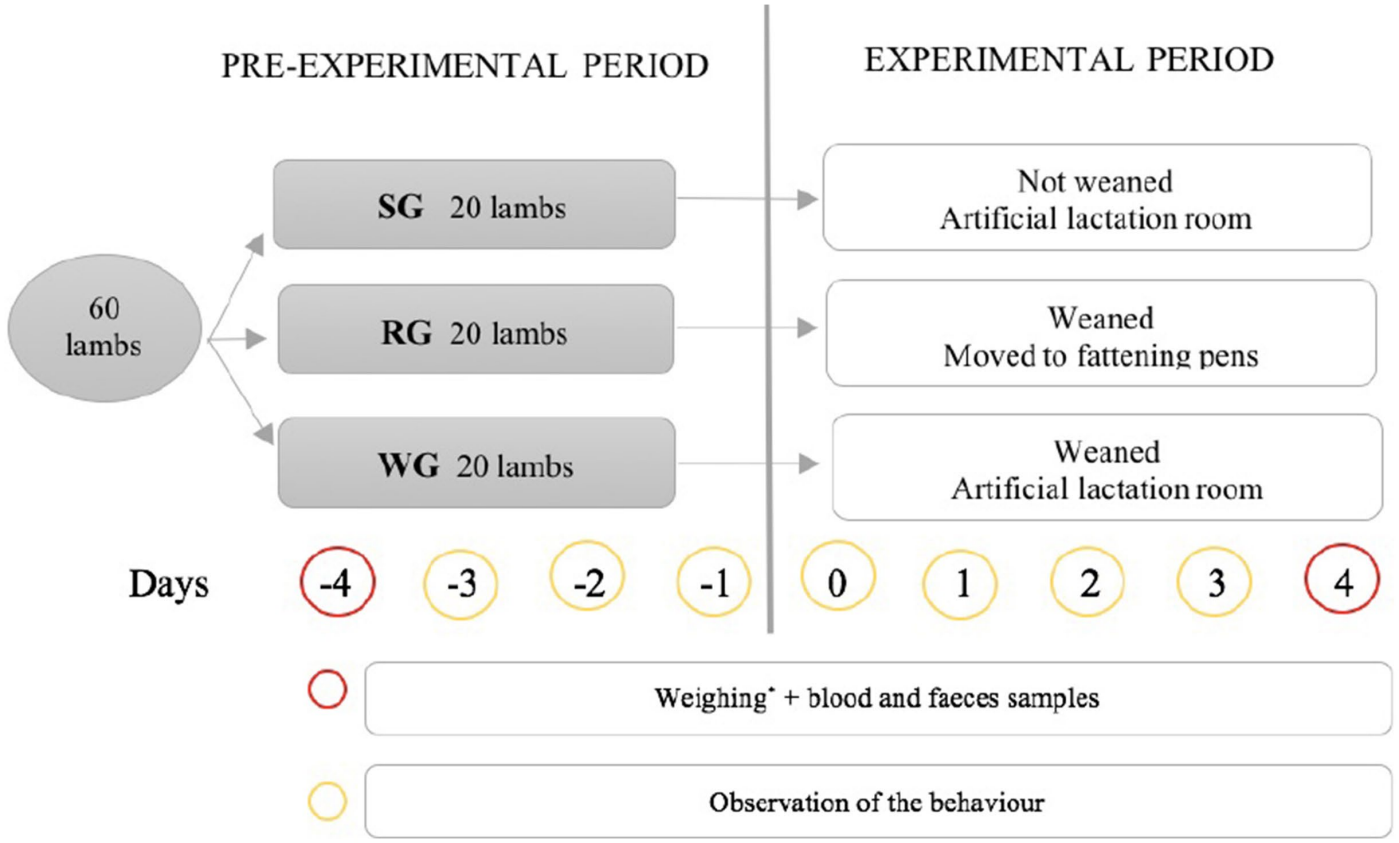
Experimental design. SG, suckling group; RG, rehoused group; WG, weaned group. *Weighing controls were also done on days −4, 0, 4, 11, 18, 25, and 31 of the experiment.

The schedule of the different actions carried out in the different groups can be consulted in Figure 1.

#### 2.2.1 Behavior variables

The observation of the behavior of each group of animals was carried out for 7 consecutive days, from 3 days before weaning to 3 days after it. These observations were done by the researchers from the distance and without having contact with the individuals (so that there were no effects of the observers on the lambs). The observation interval was divided into two periods, one in the morning and another in the afternoon. Each of the observation periods lasted 3 h, from 8:30 to 11:30 (included both) in the morning and from 15:30 to 18:30 in the afternoon, avoiding central hours in which operators of the farm cleaned feed or water troughs or add straw to the beddings.

The data or observations were taken every 15 min and during 1 min, during which the group of individuals was observed and the behavior parameters were recorded: whether they were standing (an animal was considered to develop this behavior when it was immobile with all 4 limbs touching the ground), walking (animal in motion with any of its limbs, to move from one place to another), eating (animal taking food from the feeder to its mouth, be it straw, from the trough or from the bed, or concentrate feed; in the case of the group of suckling lambs it was also considered when lambs used the milk replacer machine), ruminating (animal that carries out the action of regurgitating digestive content to its mouth, chewing it and swallowing it) and vocalizing (animal that bleats). Animals could show more than one behavior during one observation. The total observations per observation period was 13.

For each observation period and animal and in each of the experimental treatments, the following variables were recorded: (i) Frequency of times that an animal was standing (n), (ii) walking (n), (iii) eating (n), (iv) ruminating (n), and (v) vocalizing (n).

#### 2.2.2 Lamb weight yields

The lamb weight was controlled from before the weaning (−4 day of the experiment) to 31st day after the weaning.

The weighing of the animals (kg) was manually carried out using a digital dynamometer model SBS-KW-300AG (Steinberg^®^, Berlin, Germany) which allowed it to be moved between pens to facilitate the recording task. Lambs were weighed by using a digital dynamometer. Once a precise reading was obtained, it was recorded along with the number of the weighed individual, which was marked and released back with the rest of the animals. These weighing were performed on days −4, 0, 4, 11, 18, 25, and 31 of the experiment, early in the morning.

#### 2.2.3 Blood and serum parameters and count of *E. coli* colonies from lamb feces

On days −4 and + 4, two blood samples from jugular vein from each lamb were extracted by using vacuum tubes system (one with BD Vacutainer^®^ Lithium Heparin Tubes and one with BD Vacutainer^®^ Rapid Serum Tubes, Plymouth, United Kingdom). One blood sample was used to determine blood parameters at day −4 and at day +4: White Blood Cell count (WBC); Red Blood Cell (RBC); Hemoglobin (HGB); Hematocrit level (HCT); Mean Corpuscular Volume (MCV); Red Cell Distribution Width (RDW); Neutrophils count (NEUT); Lymphocytes count (LYMPH); Monocytes count (MONO); Eosinophils count (EOS) and Basophils count (BASO). Hematologic analysis was performed on the Advia 120/2120 (Siemens Healthcare Diagnostics GmbH, Berlin, Germany) by flow cytometry, which is widely used nowadays (20, 21).

The sample without anticoagulant was used to determine cortisol, C-reactive protein (CRP) and haptoblobin (HAPTO) from the serum. The method to determine cortisol was competitive ELISA, using the Salivary Cortisol ELISA kit (ref. SLV-2930) from DRG Instruments (DRG Instruments, Marburg, Germany) (22, 23). The method to determine CRP was a sandwich ELISA, using the Sheep C-Reactive Protein (CRP) ELISA kit from Life Diagnostics (catalog number CRP-12) (24). The method to determine haptoglobin was the colorimetric hemoglobin binding method (24). The reagent used for this parameter is called Haptoglobin Colorimetric Assay” PHASETM RANGE, manufactured by Tridelta Development Limited ® (Ireland).

On days −4 and + 4, one feces sample from each lamb were extracted by direct manual collection from the animal’s rectum. The processing of the samples consisted of their cultivation in MacConkey Agar culture medium (MacCONKEY No. 2 AGAR, Scharlau^®^) for the counting of colonies compatible with *E. coli*, and their isolation and typing (15, 25, 26).

The counted colonies were reseeded on EMB agar [EOSIN METHYLENE BLUE AGAR (EMB AGAR), Scharlau^®^], identifying colonies compatible with *E. coli* and allowing them to be counted (25).

On the other hand, four of the colonies counted in each sample were sown on TSA Agar [TRYPTIC SOY AGAR (TSA) (Eur. Pharm.), Scharlau^®^] obtaining pure cultures. Subsequently, these colonies were used to type the E-coli strains, using API 20-E galleries (API^®^ ID range of galleries, BIOMÉRIEUX).

### 2.3 Statistical analysis

*E. coli* counts were transformed (logEcoli = log10Ecoli) to achieve normality of their distribution.

To know the effect of the treatments applied to the lambs (rehoused group, weaned group and suckling group) on the behavioral variables (standing, walking, eating, ruminating and vocalizing) a linear mixed model (Proc GLIMMIX, SAS, 9.2, 2012) was used. Additionally to the treatment applied, the fixed effects also included the day (−3, −2, −1, 0, 1, 2, and 3). The interaction of these both effects was not significant and not included in the model. In this model, to consider repeated recordings throughout the study in the same lamb, this was considered a random term and a compound symmetry covariance structure was applied.

To know the effect of the treatments applied to the lambs (rehoused group, weaned group and suckling group) on the hematology, serum blood and feces variables of the lambs a linear mixed model (Proc GLIMMIX, SAS, 9.2, 2012) was used. Additionally to the treatment applied, the day of sampling was included in the model (−4 and 4). In this model, to consider repeated recordings throughout the study in the same lamb, this was considered a random term and a compound symmetry covariance structure was applied.

To know the effect of the treatments (rehoused group, weaned group and suckling group) on the weight yield of the lambs a linear mixed model (Proc GLIMMIX, SAS, 9.2, 2012) was used. Additionally to the treatment, the day of the weighing was included in the model (−4, 0, 4, 11, 18, 25, and 31) and the interaction of both effects. In this model, to consider repeated recordings throughout the study in the same lamb, this was considered a random term and a compound symmetry covariance structure was applied.

## 3 Results

### 3.1 Results of behavior

**Figure 2:**
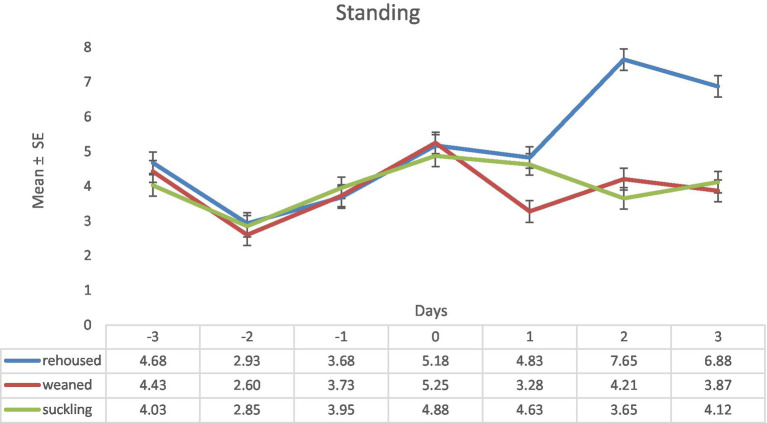
Results (mean ± standard error) of the variable standing for each lamb on each observation period in the three study groups (Standard error = 0.31).

Regarding the variable standing during the pre-experimental period there were no differences between the three groups of lambs. These differences were maintained during the beginning of the experimental period (day 0) but in the day 1, the weaned lambs showed lower values than the groups of suckling and rehoused lambs. During the days 2 and 3 the rehoused lambs showed higher values of this variable than the other two groups. Maximum differences were found on day two (rehoused: 7.65 ± 0.31 vs. suckling: 3.65 ± 0.31; Figure 2).

**Figure 3:**
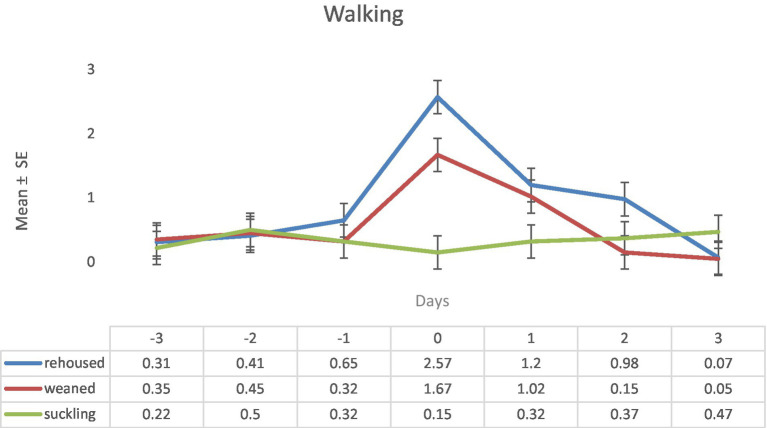
Results (mean ± standard error) of the variable walking for each lamb on each observation period in the three study groups (Standard error = 0.26).

Respect the variable walking, as expected, there were no differences up to the day 0 in which the rehoused lambs showed higher values of this variable than the group of weaned lambs and, at the same time, the weaned lambs showed higher values than the suckling lambs. On this day zero maximum differences were found (rehoused: 2.57 ± 0.26 vs. suckling: 0.15 ± 0.26). During the day 1 the differences, respect to day 0, weaned lambs and rehoused lambs significatively decreased their values of the variable walking without differences between both groups. Again, these two groups showed similar higher values of this variable than the suckling lambs, although the magnitude of the differences decreased. During the day 2, the rehoused lambs maintained the values of the day one. On the other hand, the weaned lambs decreased their values reaching similar values as the suckling lambs. On the day 3, rehoused lambs similarly to weaned lambs on the previous day decreased the values of this variable so at the end of the experiment the three groups of lambs showed similar values (Figure 3).

**Figure 4:**
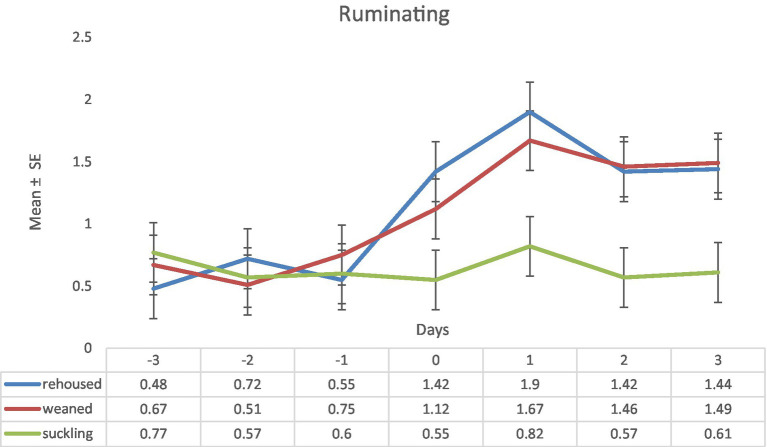
Results (mean ± standard error) of the variable ruminating for each lamb on each observation period in the three study groups (Standard error = 0.24).

Regarding the variable ruminating on days −3 to −1, as expected, there were no differences between groups. On the day 0, there was an increase of this behavior in the groups of weaned and rehoused lambs, while the group of suckling lambs maintained the values of this variable. During the day 1, respect to the day 0, weaned and rehoused lambs increased significatively this behavior and in the days 2 and 3 continued with similar higher values than suckling lambs. Maximum differences were found on day one (rehoused: 1.9 ± 0.24 vs. suckling: 0.82 ± 0.24; Figure 4).

**Figure 5:**
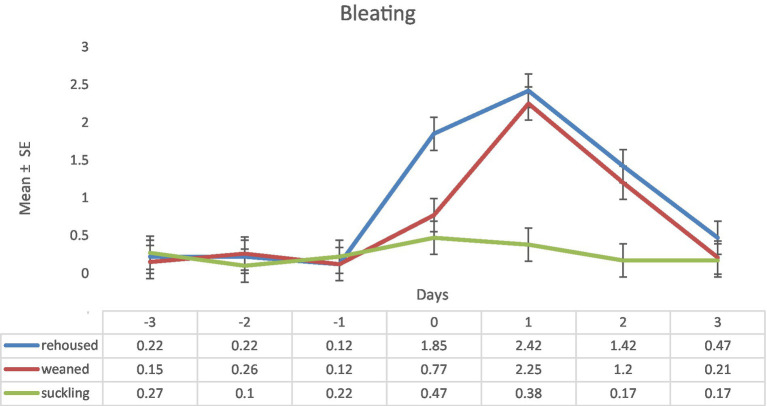
Results (mean ± standard error) of the variable bleating for each lamb on each observation period in the three study groups (Standard error = 0.22).

Respect to the variable bleating, there were no differences between groups of lambs during the pre-experimental period. On the day 0, the rehoused lambs showed an increase of this behavior. The weaned lambs also increased this behavior but did not reach the values of the rehoused lambs. On the day 1, respect to day 0, increased, even more, this behavior. Weaned lambs also increased the values of bleating, reaching the values of the rehoused lambs. Maximum differences were found on day one (rehoused: 2.42 ± 0.22 vs. suckling: 0.38 ± 0.22). On day 2, both groups experienced a decrease of this behavior but still with higher values than the suckling lambs. On day 3, weaned and rehoused lambs decreased the bleating behavior to the same level of the suckling lambs, which maintained similar values along the pre-experimental and the experimental periods (Figure 5).

**Figure 6:**
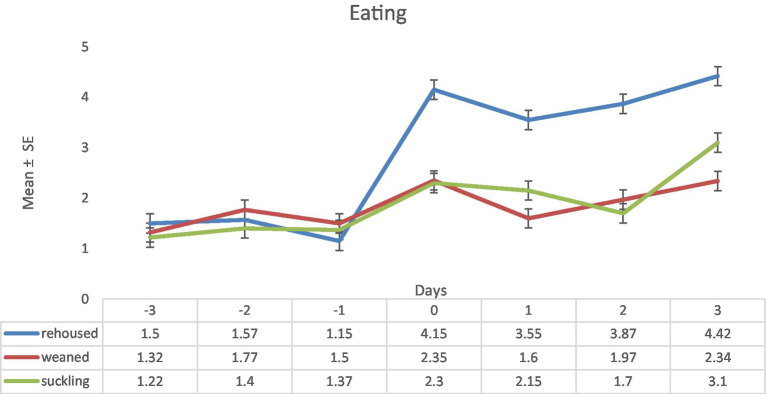
Results (mean ± standard error) of the variable eating for each lamb on each observation period in the three study groups (Standard error = 0.19).

Regarding the variable eating, rehoused lambs increased this behavior from the day 0 of the experiment, just after the change of conditions and maintained similar values of this variable along the experimental period. On day zero the maximum differences found were: rehoused: 4.15 ± 0.19 vs. suckling: 2.3 ± 0.19. Weaned and suckling lambs showed slightly higher average values of this variable during the experimental period than in the peexperimental period with significative daily variations that even entailed significative variation between both groups on days 1 and 3. On day three there were differences between the three groups (rehoused: 4.42 ± 0.19 vs. weaned: 2.34 ± 0.19 vs. suckling: 3.1 ± 0.19; Figure 6).

### 3.2 Results of lamb weight yields

The weight of the suckling lambs followed a linear pattern over time. Both the rehoused group and weaned group exhibited significant differences compared to the suckling group in terms of weight on days 4 and 11 of the experiment, during which both groups experienced a slowdown in growth, even showing weight loss. However, these statistical differences in weight disappeared by day 18 of the experiment. In an additional second statistical analysis, it was demonstrated that the average daily weight gain during days 4–11 was higher in the weaned group compared to the other two groups (*p* < 0.05), and it was also higher in the rehoused group than in the suckling group of lambs (p < 0.05). However, after day 18, there were no significant differences in weight or average daily gain between the three groups (Table 1).

**Table 1:** Results (mean ± SE) of the lamb weight yields in the three groups of the study during pre-experimental and experimental period.

	Pre-experimental period		Experimental period				
Group	Weight D -4 (kg)	Weight D 0 (kg)	Weight D 4 (kg)	Weight D 11 (kg)	Weight D 18 (kg)	Weight D 25 (kg)	Weight D 31 (kg)
WG	16.35 ± 0.44	17.87 ± 0.47	17.55 ± 0.55^a^	19.19 ± 0.59^a^	21.18 ± 0.75	23.11 ± 0.79	24.95 ± 0.89
RG	16.37 ± 0.44	17.37 ± 0.47	17.11 ± 0.55^a^	19.11 ± 0.59^a^	20.84 ± 0.75	22.94 ± 0.79	24.73 ± 0.89
SG	16.8 ± 0.44	17.99 ± 0.47	19.72 ± 0.55^b^	20.69 ± 0.59^b^	22.41 ± 0.75	24.4 ± 0.79	25.24 ± 0.89

SG, suckling group; RG, rehoused group; WG, weaned group. Values in the same line with different letters (a, b) differ at *p* < 0.05.

### 3.3 Results of blood and serum parameters and count of *E. coli* colonies from lamb feces

The results of most of the variables recorded through blood sampling during the pre-experimental sampling (4 days before the change of conditions) showed no significant differences among the three groups of lambs. The only variable that exhibited differences during the pre-experimental sampling was the eosinophil count, wherein the group of lambs that were subsequently rehoused displayed higher values than the lambs that were subsequently weaned, and both these groups showed higher values than the group that remained in the same barn and continued to be fed with milk replacer.

During the experimental period, there were minimal changes observed between the groups. Once again, most of the variables showed no significant differences among the groups. However, the red blood cell count (RBC) exhibited higher values in the two groups of lambs that remained in the same barn (weaned and suckling lambs) compared to those that were relocated to another barn. Additionally, the hemoglobin level (HGB) was higher in the group of suckling lambs than in the group of rehoused lambs. Weaned lambs displayed intermediate HGB values with no statistically significant differences compared to the other two groups. On the other hand, the differences observed in the eosinophil count were maintained in the experimental period, appearing the same differences as during the pre-experimental period (Table 2).

**Table 2:** Results (mean ± SE) of blood cells counts in the three groups of the study during both pre-experimental and experimental sampling.

Variable	Rehoused	Weaned	Suckling	SL
WBC_P_ (10^3^cells/μL)	7.72 ± 0.75	6.63 ± 0.75	6.71 ± 0.75	ns
WBC_E_ (10^3^cells/μL)	6.64 ± 0.41	6.88 ± 0.41	6.38 ± 0.41	ns
RBC_P_ (10^6^cells/μL)	9.75 ± 0.43	9.96 ± 0.43	10.34 ± 0.43	ns
RBC_E_ (10^6^cells/μL)	10.34 ± 0.22 b	11.51 ± 0.22 a	11.94 ± 0.22 a	<0.01
HGB_P_ (g/dL)	9.78 ± 0.39	9.85 ± 0.39	10.01 ± 0.39	ns
HGB_E_ (g/dL)	9.81 ± 0.17 b	9.98 ± 0.17 ab	10.31 ± 0.17 a	<0.01
HCT_P_ (%)	31.02 ± 0.78	30.54 ± 0.78	31.69 ± 0.78	ns
HCT_E_ (%)	32.79 ± 0.54	32.42 ± 2.3	37.73 ± 2.3	ns
MCV_P_ (fl)	30.15 ± 0.68	30.75 ± 0.68	30.78 ± 0.68	ns
MCV_E_ (fl)	28.71 ± 0.58	29.13 ± 0.58	28.74 ± 0.58	ns
RDW_P_ (%)	20.73 ± 0.53	20.21 ± 0.53	20.02 ± 0.53	ns
RDW_E_ (%)	20.15 ± 0.47	20.01 ± 0.47	19.46 ± 0.47	ns
NEUT_P_ (10^3^cells/μL)	2.90 ± 0.59	2.97 ± 0.59	2.94 ± 0.59	ns
NEUT_E_ (10^3^cells/μL)	2.24 ± 0.31	2.43 ± 0.31	2.14 ± 0.31	ns
LYMPH_P_ (10^3^cells/μL)	3.32 ± 0.36	2.82 ± 0.36	2.77 ± 0.36	ns
LYMPH_E_ (10^3^cells/μL)	3.24 ± 0.21 b	3.23 ± 0.21	3.49 ± 0.21	ns
MONO_P_ (10^3^cells/μL)	0.43 ± 0.08	0.31 ± 0.08	0.38 ± 0.08	ns
MONO_E_ (10^3^cells/μL)	0.29 ± 0.04	0.32 ± 0.04	0.29 ± 0.04	ns
EOS_P_ (10^3^cells/μL)	0.71 ± 0.06 a	0.54 ± 0.06 b	0.29 ± 0.06 c	<0.01
EOS_E_ (10^3^cells/μL)	1.03 ± 0.07 a	0.71 ± 0.07 b	0.16 ± 0.07 c	<0.01
BASO_P_ (10^3^cells/μL)	0.09 ± 0.01	0.07 ± 0.01	0.09 ± 0.01	ns
BASO_E_ (10^3^cells/μL)	0.08 ± 0.01	0.08 ± 0.01	0.08 ± 0.01	ns

SL, Significance level; ns, not significant; WBC_p_, White Blood Cell pre-experimental; WBC_e_, White Blood Cell experimental; RBC_P_, Red Blood Cell pre-experimental; RBC_E_, Red Blood Cell experimental; HGB_P_, Hemoglobin pre-experimental; HGB_E_, Hemoglobin experimental; HCT_P_, Hematocrit level pre-experimental; HCT_E_, Hematocrit level experimental; MCV_P_, Mean Corpuscular Volume pre-experimental; MCV_E_, Mean Corpuscular Volume experimental; RDW_P_, Red Cell Distribution Width pre-experimental; RDW_E_, Red Cell Distribution Width experimental; NEUT_P_, Neutrophils count pre-experimental; NEUT_E_, Neutrophils count experimental; LYMPH_P_, Lymphocytes count pre-experimental; LYMPH_E_, Lymphocytes count experimental; MONO_P_, Monocytes count pre-experimental; MONO_E_, Monocytes count experimental; EOS_P_, Eosinophils count pre-experimental; EOS_E_, Eosinophils count experimental; BASO_P_, Basophils count pre-experimental; BASO_E_, Basophils count experimental. Values in the same line with different letters (a, b, c) differ at *p* < 0.05.

During both the pre-experimental and experimental period, no significant differences were observed between the three groups of lambs in any of the variables measured on blood serum and feces. The cortisol levels remained relatively stable in all groups throughout both periods, with no significant changes. The levels of C-reactive protein (CRP) did not vary significantly among the experimental groups during either period. The haptoglobin levels also showed no significant differences between the groups in both the pre-experimental and experimental periods (Table 3). Likewise, the log *E. coli* counts in feces did not exhibit significant variations between the groups during either period (Table 4).

**Table 3:** Results (mean ± SE) of serum stress indicators in the three groups of the study during both pre-experimental and experimental sampling.

	Rehoused	Weaned	Sucking	SL
CORTISOL_P_ (ng/ml)	2.27 ± 0.31	1.69 ± 0.31	1.97 ± 0.31	ns
CORTISOL_E_ (ng/ml)	2.95 ± 0.59	3.71 ± 0.59	2.79 ± 0.59	ns
CRP_P_ (μg/mL)	51.29 ± 3.11	47.99 ± 3.11	47.53 ± 3.11	ns
CRP_E_ (μg/mL)	53.73 ± 3.07	45.55 ± 3.07	52.19 ± 3.07	ns
HAPTO_P_ (mg/mL)	0.34 ± 0.07	0.33 ± 0.07	0.36 ± 0.07	ns
HAPTO_E_ (mg/mL)	0.53 ± 0.11	0.52 ± 0.11	0.44 ± 0.11	ns

CORTISOLP, Cortisol pre-experimental; CORTISOLE, Cortisol experimental; CRPP, C-reactive protein pre-experimental; CRPE, C-reactive protein experimental; HAPTOP, haptoblobin pre-experimental; HAPTOE, haptoblobin experimental; SL, Significance level; ns, not significant.

**Table 4:** Results (mean ± SE) of count of *E. coli* colonies from lamb feces in the three groups of the study during both pre-experimental and experimental sampling.

	Rehoused	Weaned	Sucking	SL
LOG Ecoli_P_ (UFC/g)	5.93 ± 0.46	5.09 ± 0.46	5.51 ± 0.46	ns
LOG Ecoli_E_ (UFC/g)	5.09 ± 0.69	5.48 ± 0.69	6.17 ± 0.69	ns

LOG EcoliP, *E. coli* pre-experimental; LOG EcoliE, *E. coli* experimental; SL, significance level; ns, not significant.

## 4 Discussion

Understanding the behavioral and physiological responses of lambs during weaning can help identify potential stressors and their effects on health and growth (27). Lambs raised on artificial milk have nutritional and welfare conditions that can impact their development and well-being. Studies on this subject can inform best practices for weaning management to minimize distress and enhance animal welfare. By addressing these factors, we can improve the overall husbandry practices, ensuring healthier animals and more sustainable farming operations.

### 4.1 Behavior

During the pre-experimental period (days −3 to 0), as expected, there were no differences observed between the three groups of lambs, displaying the typical behavior of artificially fed lambs, with short periods of suckling and long periods of resting (low periods of standing and walking) (28). Lambs of this age (34 ± 3 days old) tend to increase amount of milk per feeding reducing suckling frequency and due that lambs were used to presence of human to help them to be fed, during daily maintenance of the artificial milk machine (this daily period was not used for the results) most of the lambs tend to suck during this daily task resulting in reduced activity periods during the rest of the day. However, during the experimental period, the behavior of the lambs was differently affected based on the treatment applied, leading to significant differences between the groups according to changes in location, feeding, or a combination of both.

Several studies have investigated the behavioral effects of weaning after natural nursing in suckling lambs. These investigations have shown that lambs experience an increase in vocalizations (29) and engage in more standing, walking, and pacing activities after weaning (30, 31). The findings of our present study align with these previous results. The lambs in our study, which were rehoused and experienced a change in their feed and environment, demonstrated higher levels of standing, walking, eating, and bleating. Interestingly, contrary to adult ewes in Cockram et al. (32), the lambs in our study exhibited an increase in ruminating behavior. This could be explained by their preference for milk replacer during the pre-experimental period, leading to greater interest in solid feed when milk replacer was no longer available during the experimental period, always considering that solid feed was not a novelty for them since it was available from their birth. It could be thought that stress also reduces rumination, but it is true that they had the feed and straw during the pre-experimental period, so it was not unknown to them. As demonstrated in previous articles the transition to solid feed stimulates the rumen function development and the ruminating behavior in ruminants (33, 34).

Furthermore, it was observed that the change in location was responsible for the increase in standing and eating behaviors, as weaned lambs in the same location but with different feed exhibited similar values to suckling lambs in these variables. Similarly, weaned lambs displayed intermediate values between rehoused lambs and suckling lambs in walking and bleating, indicating synergistic stressing effects of both the change in feed and the change in location. These findings highlight the importance of considering both factors when studying the behavioral responses of lambs after weaning. Feeding changes without a change of location are a fully satisfactory solution, especially in bleating, which can be considered the most representative variable of an alteration in animal welfare.

### 4.2 Lamb weight yields

Weaning after lactation with the mother can initially lead to a slowdown in the growth rate of lambs due to the change in diet and the potential stress associated with separation from their mothers. However, as they adapt to their new diet, their growth rate increases again (35). The present study also demonstrated that weaning after feeding with artificial milk results in a growth slowdown. This outcome can be primarily attributed to the change in feed, and not to the change of location, as there were no differences in growth between the lambs that remained in the same barn but were fed concentrate and straw. The switch in feed led to a halt in the animal’s growth, followed by compensatory growth over the subsequent 7 days. It is noteworthy that rehoused lambs exhibited higher levels of eating and ruminating behaviors, indicating increased feed intake. However, these lambs also displayed higher levels of bleating, standing, and walking, which suggests they utilized the nutrients from the feed to sustain a higher level of activity rather than promoting growth (36).

### 4.3 Blood and serum parameters and count of *E. coli* colonies from lamb feces

The hematological values of the three groups of healthy lambs in the study are similar to those described in the studies by Navarro et al. (7) and Bórnez et al. (37) and within the intervals of healthy animals described by Jones and Allison (38) and Lepherd et al. (39).

The three groups of lambs in our study experienced changes in the values of certain hematological parameters over the course of sampling, which took place over a period of 8 days. Specifically, there was an increase in the values of RBC, HGB, and HCT. These changes in blood parameters could be indicative of physiological adaptations that occur during the weaning process and the transition from a milk-based diet to solid feed. These findings align with the results reported by Bórnez et al. (37) in their study of suckling and light lambs, confirming a consistent upward trend in these parameters as the lambs age. Additionally, values of RBC in the second sampling were higher in weaned and suckling lambs than in rehoused lambs. Our rehoused lambs were transported to a barn which was 200 m far from the original barn. This result could agree with the results of previous studies of Bórnez et al. (37) and Navarro et al. (7) who described that even short trips (30 min and 1 h respectively) next to handling, uploading and unloading were enough to produce a release free radical that induced erythrocyte lysis. Our results would confirm this hypothesis as weaned lambs, which stayed in the same barn but with a change in the feed, showed similar values compared to suckling lambs. Interestingly, HGB of weaned lambs showed intermediate values between rehoused lambs and suckling lambs. This fact could point to some additive effects of change of location and change of feed in the levels of HGB. Values of eosinophils during the pre-experimental period were different between the three experimental groups (rehoused > weaned > suckling). These differences were maintained after the experimental period but interestingly, while values of the rehoused and weaned lambs increased, the values of the suckling lambs decreased. Elevation of eosinophils are usually employed to indicate parasitology diseases (40, 41) and some authors have registered eosinopenia as an stress indicator (42). In our study the difference found between groups can be explained by some undetermined factor happened before the beginning of the study as it can be observed in the preexperimental values of this variable. In this sense, other studies have not found a relation between stress and values of eosinophils (43). When lambs are transported to a feedlot, not being fattened in the same location where they were born, it has been shown that handling and transport directly influence the factors indicative of stress in the animals (12), in our study the fattening occurred on the same farm, but 200 meters away, that could explain why the stress values are not so high.

Finally, after the results found on this study, the research on new methods and alternatives to reduce the stress of lambs during weaning must be subject of study. Although the compensatory growth found in the weaned and the rehoused lambs and the non-existing differences in fecal and serum stress indicators, the changes of behavior and the delay in the growth of the lambs are, by themselves, indicators of alterations in the animal welfare that must also be considered from an ethical point of view.

## 5 Conclusion

Lambs weaned after milk replacer feeding subjected to changes in feeding and location demonstrated higher levels of activity in several behavioral parameters, with different responses found depending on the change in feeding and/or location. After weaning, a temporary delay in the growth of the lambs fed with milk replacer was observed. This delay was mainly due to the change in feeding rather than the change in location. These findings highlight the importance of considering both factors when studying the behavioral responses of lambs after weaning as a possible assessment of their welfare. On the other hand, serum and fecal stress indicators did not show significant differences between the three groups studied. As it has been showed in this study and in others, weaning from artificial milk replacers represents a challenge for the animal welfare of lambs, therefore more studies must be carried out to have a deeper knowledge of these factors.

## Data Availability

The original contributions presented in the study are included in the article/supplementary material, further inquiries can be directed to the corresponding author.
